# Photodynamic therapy in the treatment of aggressive periodontitis: A systematic review

**DOI:** 10.4317/medoral.21046

**Published:** 2015-11-22

**Authors:** Georgios-Sokratis Chatzopoulos, Aikaterini-Ellisavet Doufexi

**Affiliations:** 1 DDS. Advanced Education Program in Periodontology, University of Minnesota, 515 Delaware Street SE, Minneapolis, MN 55455, USA; 2 DDS, PhD. Private practice limited to periodontics and implant dentistry

## Abstract

**Background:**

Aggressive periodontitis (AgP) is a severe form of periodontal diseases with rapid destruction of the supporting bone around teeth. The efficacy of PDT in suppressing periodontal pathogens may be crucial in adopting new protocols for the treatment of AgP. Thus, the aim of this systematic review was to investigate the possible role of PDT in the treatment of AgP as an adjunctive therapy or monotherapy.

**Material and Methods:**

A systematic search of the literature was performed. Additionally, the references from all the selected full-text studies were searched for relevant articles. Two reviewers screened independently titles and abstracts or full text copies. Quality assessment of all the included studies was held.

**Results:**

Initial screening of electronic databases yielded 418 potentially relevant publications. After screening of the titles and full-text examination, five studies were included in the systematic review. Four publications evaluated the effects of PDT adjunctive to SRP in patients with AgP: two of them compared the clinical outcomes of SRP and PDT with a control group that received therapy with SRP and antibiotics (metronidazole and amoxicillin); two publications included SRP and PDT in the test group, and SRP alone in the control group. In one study, PDT was tested as a monotherapy compared with SRP alone.

**Conclusions:**

Within the limitations of this review, PDT may exhibit a beneficial role in the therapy of aggressive periodontitis after repeated applications. In the future, more methodologically sound, long-term randomized clinical trials are needed to be conducted.

**Key words:**Photodynamic therapy, periodontitis, systematic review.

## Introduction

Aggressive periodontitis (AgP) is a severe form of periodontal diseases with rapid destruction of the supporting bone around teeth. Bone loss and destruction of periodontal attachment lead to tooth loss (1). The microbiological background of AgP has been identified by several studies including species such as *A. actinomycetemcomitans, P. gingivalis, Capnocytophaga species, Eikenella corrodens, P. intermedia, and Campylobacter rectus* (2). AgP usually affects young individuals, and the prevalence among young subjects under 35 years old ranges between 1% and 15%, depending on the age (3). Nibali *et al*. concluded after a meta-analysis of the existing studies that the average tooth loss for patients with AgP is approximately 0.09 (95% C.I. = 0.06-0.16) per year. In contrast, individuals with unspecified or chronic periodontitis are expected to lose 0.10 to 0.30 teeth per year (4).

The initial therapy of AgP aims to eliminate the bacterial load of periodontal pockets, following the same methods with the therapy of chronic periodontitis. Scaling and root planing (SRP) consists the first step of the treatment with adequate results during six months after therapy. After that period of time, albeit the active periodontal maintenance program, disease progression has been recorded (5). However, the treatment in the majority of patients with AgP is challenging and SRP does not diminish effectively the prevalence of the bacteria (6). The effectiveness of the adjunctive systemic antibiotics was examined in a systematic review conducted by Keestra *et al*. (7). Systemic antibiotics in combination with SRP showed a significant effect for the treatment of AgP in comparison with SRP alone. The combination of metronidazole and amoxicillin plays a key role in the treatment outcome: 0.51 ± 0.38 mm probing pocket depth reduction; 0.46 ± 0.37 mm clinical attachment level gain; 12.76 ± 10.35% bleeding on probing reduction at 12 months after therapy (7).

Photodynamic therapy (PDT) has emerged recently as a new noninvasive treatment modality. A photosensitizer (or photoactivatable agent) that absorbs light, can be absorbed in a target tissue. Free radicals and singlet oxygen molecules are produced by multiple reactions originated from the interaction of the photosensitizer with a light of a proper wavelength and oxygen (8). In dentistry, PDT has been proposed as a novel disinfection method that it could be a potential treatment for several infectious diseases by eradicating microorganisms. This use is often mentioned as antimicrobial PDT (a-PDT). Due to the high antibacterial potential, PDT has been proposed in the treatment of chronic periodontitis, peri-implantitis and endodontic infections (9-11). However, there is still insufficient evidence to support the superiority of PDT in periodontal and peri-implantitis treatment compared to scaling and root planing alone or as an adjunct (9,12-15).

The effects of PDT on the treatment of chronic periodontitis have been systematically reviewed, but previous systematic reviews have not examined the effectiveness of that treatment modality in AgP. The efficacy of PDT in suppressing periodontal pathogens may be crucial in adopting new protocols for the treatment of AgP. Thus, the aim of this systematic review was to investigate the possible role of PDT in the treatment of AgP as an adjunctive therapy or mono therapy.

## Material and Methods

This systematic review was conducted based on QUOROM statement guidelines, as described by Moher *et al*. (16).

Focused questions

The following focused questions have been utilized to identify the possible role of PDT in AgP treatment:

1) “Do PDT combined with SRP vs. SRP alone in untreated AgP patients have an additional effect on the clinical outcomes?”

2) “Do PDT combined with SRP vs. SRP with antibiotic therapy in untreated AgP patients have an additional effect on the clinical outcomes?”

3) “What is the efficacy of PDT, when compared with SRP alone in the treatment of AgP?”

Search strategy

The following databases were searched from their earliest records until March 16th, 2015: Cochrane Central Register of Controlled Trials and other evidence-based medicine reviews (The Cochrane Library), MEDLINE-Pubmed, SCOPUS and Science Direct. The following terms were used during the search (“Periodontitis”[Mesh] OR “Aggressive Periodontitis”[Mesh] OR “Periodontal Diseases”[Mesh] OR “Periodontal Pocket”[Mesh] OR “Periodontal Attachment Loss”[Mesh] OR “Tooth Mobility”[Mesh] OR periodontitis OR periodontal disease* OR periodontal pocket* OR attachment loss OR alveolar bone loss OR pocket depth OR clinical attachment level) AND (therapy OR treatment OR intervention) OR (periodontal non surgical treatment OR periodontal non surgical therapy OR scaling root planing OR dental scaling OR periodontal treatment OR periodontal therapy OR calculus remov* OR calculus debridement OR dental debridement OR periodontal debridement OR “Dental Scaling”[Mesh] OR “Root Planing” [Mesh] OR “Dental Prophylaxis”[Mesh] AND (“Photochemotherapy”[Mesh] OR photodynam* therapy OR photodynam* treatment OR photochem* therapy OR photodynam* OR photo therapy OR photochem* treatment). In addition, a manual search was performed of all the online issues of the following journals: Journal of Clinical Periodontology, International Journal of Periodontics and Restorative Dentistry, Journal of Periodontology, Journal of Dental Research, Journal of Periodontal Research, Periodontology 2000, Journal of Dentistry, Journal of the American Dental Association, Lasers in Medical Science, Lasers in Surgery and Medicine, Clinical Oral Investigations, Photomedicine and Laser Surgery, Photodiagnosis and Photo dynamic Therapy, Journal of Photochemistry and Photobiology B. Additionally, the references from all the selected full-text studies were searched for relevant articles.

Inclusion/Exclusion criteria

The selection for eligible studies was performed by two masked examiners aiming to reduce the potential bias from the review process. First of all, both titles and abstracts of the studies after initial search were examined for eligibility. In the second phase, all the preselected eligible studies were read in full-text independently by the examiners. Disagreements between examiners were then solved by discussion during both first and second phase. The following inclusion criteria were adopted for the studies:

1) Randomized controlled clinical trial comparing PDT vs SRP alone, PDT + SRP vs SRP alone or PDT +SRP vs SRP + antibiotics, with at least one month reevaluation.

2) The participants of the study must have been diagnosed with AgP.

3) No language restrictions included in the search strategy.

Only studies that met the previous inclusion criteria were included and they were also analyzed regarding the following exclusion criteria:

1) Individuals with systemic diseases that may affect the progression of AgP (diabetes mellitus, cardiovascular disease, cancer, immunologic disorders)

2) SRP or antibiotic therapy in the last 6 months

3) Pregnancy

4) Primary outcome of interest not analyzed.

- Outcome variables

The primary outcomes were probing pocket depth (PPD) reduction and clinical attachment level (CAL) gain. The secondary outcome was bleeding on probing (BOP) change. Changes in the primary and secondary outcomes were determined based on the baseline and the follow-up measurements.

- Data extraction

From all the included studies, the two independent examiners extracted data such as name of the first author, year of publication, origin, smoking history, study design, photosensitizer laser, laser parameters, treatment arms, number of patients, age, length of follow-up, and main conclusions. The extracted data are presented in  Table 1.

**Table 1:**
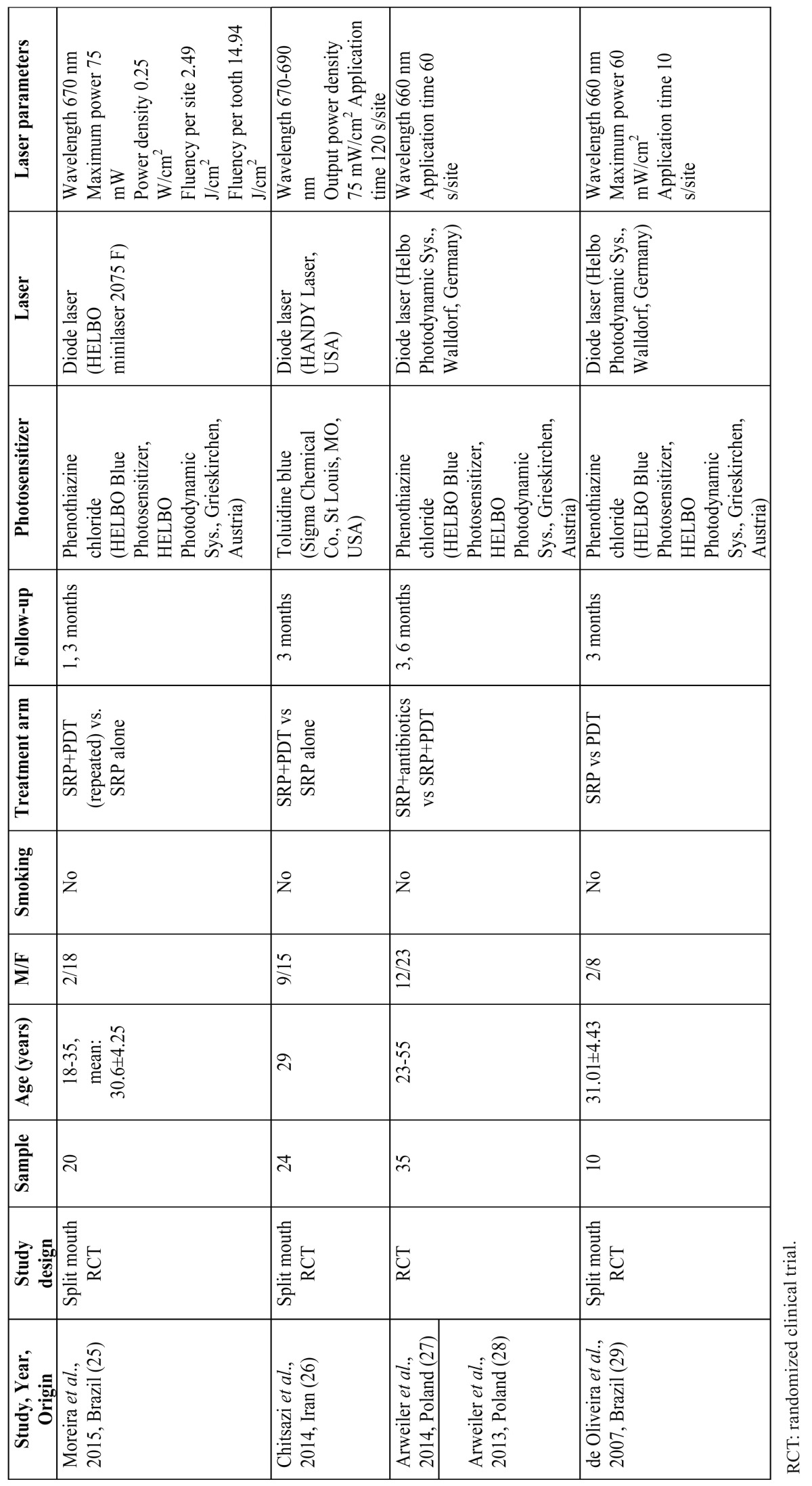
Main characteristics of the included studies.

- Quality assessment

A quality assessment of all the included studies in this systematic review was conducted in order to evaluate the adopted methodologies. It was performed independently again by the two masked examiners. Any disagreement again was resolved by discussion. The revised recommendations of the CONSORT statement were utilized, which include the following criteria: sample size calculation; randomization and allocation concealment methods; clear definition of inclusion and/or exclusion criteria; completeness of follow-up; experimental and control groups comparable at baseline for prognostic factors; presence of masking; and appropriate statistical analysis (17). When all the mentioned criteria were fulfilled, the study was determined as a low risk of bias. Moderate risk exhibited studies when one or more criteria were partly met, and risk of bias was high when one or more criteria were not met (18).

## Results

**Figure 1:**
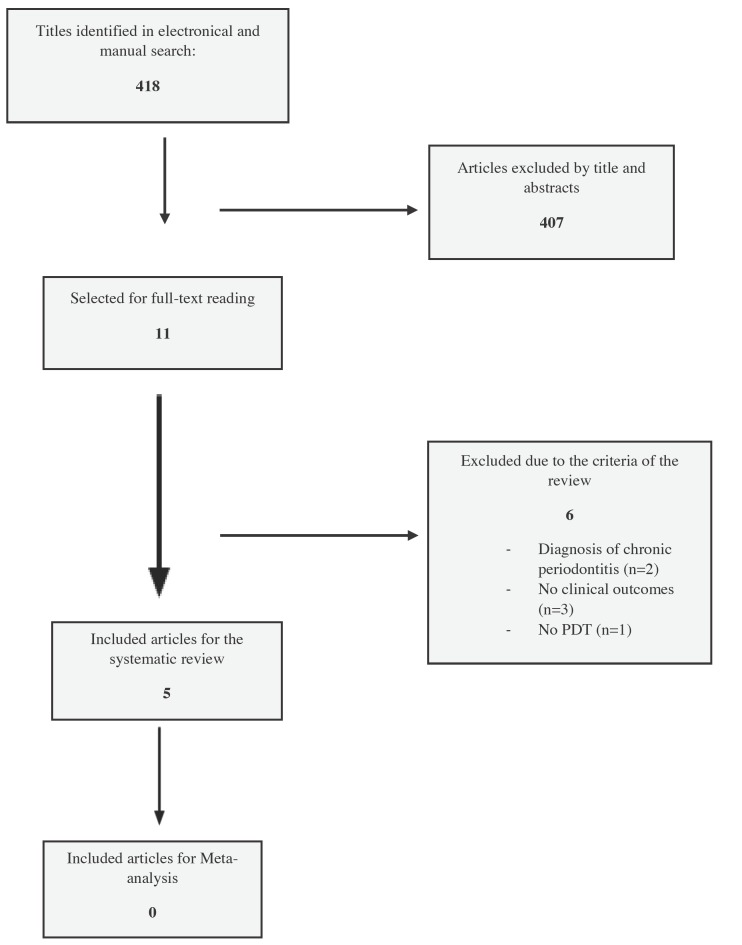
Flowchart of the search strategy.

Initial screening of electronic databases yielded 418 potentially relevant publications. Manual search of the journals did not add any other publications to the above collection. Thus, 418 publications were included in the first phase of selection. After screening of the titles and abstracts independently by the two examiners, eleven studies were retrieved for assessment of eligibility for inclusion in the systematic review (19-29). After full-text examination, six publications were excluded based on the inclusion and exclusion criteria (19-24). Screening of the reference lists of all the included full text publications did not reveal any additional relevant articles. Therefore, five studies were included in the systematic review (25-29) (Fig. 1).

- Study characteristics and risk assessment

The characteristics and the quality assessment of the included studies are presented in  Table 1, Table 2 respectively. These five included articles represented four studies that utilized different treatment arms aiming to examine the possible role of PDT in the treatment of AgP as an adjunctive therapy or mono therapy. Four publications evaluated the effects of PDT adjunctive to SRP in patients with AgP (25-28): two of them compared the clinical outcomes of SRP and PDT with a control group that received therapy with SRP and antibiotics (metronidazole and amoxicillin) (25,26); the other two publications included SRP and PDT in the test group, and SRP alone in the control group (27,28). In the last included study, PDT was tested as a monotherapy compared with SRP alone (29).

**Table 2:**
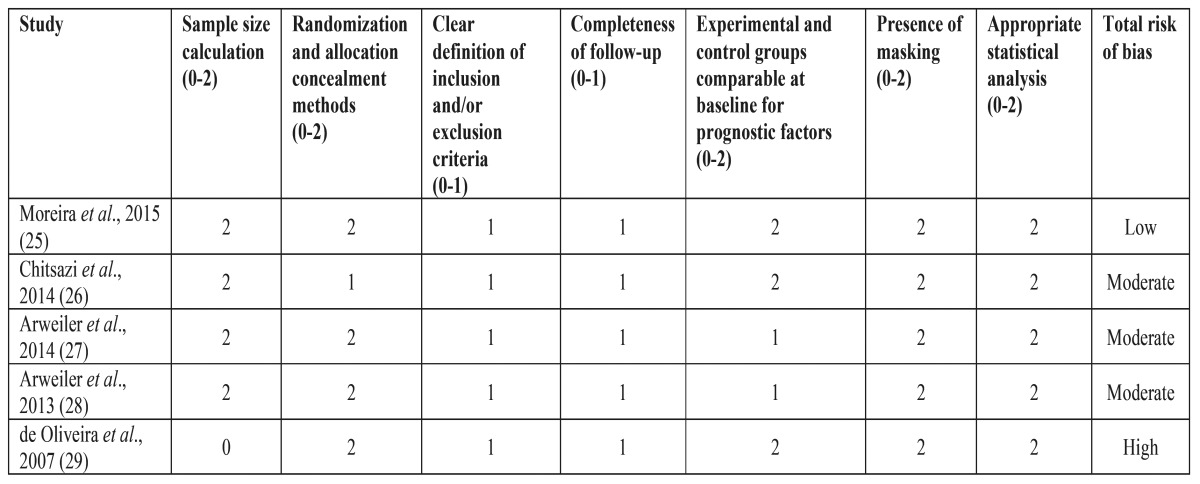
Quality assessment of the included randomized clinical trials based on CONSORT criteria [17].

- PDT + SRP vs. SRP alone

Both included studies on the effect of PDT as an adjunct to SRP in AgP cases were split mouth, double masked, randomized controlled clinical trials. In one study (25), PDT was applied at four times (baseline, 2, 7 and 14 days after therapy), whereas the other study (26) included one application of the PDT. In both publications, the included parameters were recorded at baseline and up to three months after therapy. In both included split mouth controlled studies the teeth of one maxillary quadrant were treated with PDT and SRP, while in the teeth of the contra lateral control quadrant were provided SRP alone. One study utilized phenothiazine chloride as a photo sensitizer (25), whereas the other used toluidine blue photosensitize dye (26). The diode lasers in both studies had a wavelength of 670 nm and a power of 75 mW.

Bias assessment revealed that one study had low risk of bias (25), whereas the other one moderate (26). The included publications did not allow us for a meta-analysis due to the different methodologies followed.

PDT + SRP vs. SRP + antibiotics

Two publications aimed to evaluate PDT as an alternative to systemic antibiotics in the treatment of AgP in combination with SRP (27,28). Both of them were parts of the same study presenting the assessed clinical parameters in three and six months after treatment. The study design was examiner-blind, randomized clinical trial performed in a single center. The participants were nonsmokers and the age range was 23 to 55 years. The 35 participants were included in two parallel groups of 18 (antibiotics) and 17 (PDT) in which oral hygiene instructions and SRP were provided. The phenothiazine chloride was utilized as a photosensitizer in the test group, and the diode laser demonstrated a wavelength of 660 nm. On the other hand, in the control group was prescribed antibiotic therapy with amoxicillin 375 mg and metronidazole 250 mg three times per day for seven days.

Bias assessment revealed that both studies had moderate risk of bias (27,28). The included publications did not allow us for a meta-analysis due to the lack of controlled clinical trials in this category.

- PDT vs. SRP alone

Only one study was identified during the search that aimed to examine the applicability of PDT in the treatment of AgP as a mono therapy compared to non-surgical periodontal therapy utilizing mechanical therapy (29). A split-mouth, randomized controlled clinical trial with blind examiner from Brazil included ten individuals mean aged 31.01 years with AgP. Patients were followed up for a period of three months. One randomly selected tooth was treated either with SRP or PDT, whereas in the contra lateral tooth the opposite treatment was applied. The phenothiazine chloride was selected as a photosensitizer in the PDT group in conjunction with a diode laser with a wavelength of 660 nm and 60 mW/cm2 power. Bias assessment revealed that the study exhibited high risk of bias.

Results of the included studies

The main conclusions of the included studies are presented in  Table 3.

**Table 3:**
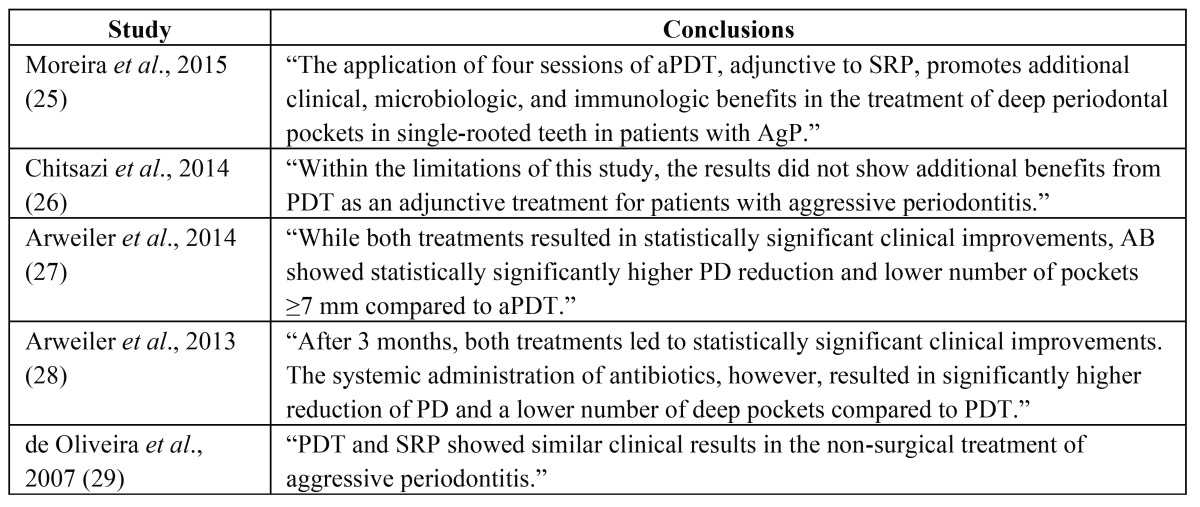
Conclusions of the included studies.

- PDT + SRP vs. SRP alone

The repeated application of PDT as an adjunct to non-surgical periodontal treatment revealed that it has clinical, microbiologic, and immunologic superiority compared to SRP alone (25). PPD reduction of moderate and deep pockets were significantly decreased in test and control group after 90 days of the treatment. However, test group exhibited a significantly higher decrease in PPD and gain in CAL of deep pockets. After 90 days, the mean differences in PPD between the two groups were 0.17 mm for moderate pockets, and 1.356 mm for deep pockets. In addition, mean differences in CAL were 0.0165 mm and 1.051 mm for moderate and deep pockets respectively. BOP three months after therapy ranged between 13.75% (test group) and 15% (control group). The residual pockets was significantly lower in the test group treated with PDT and SRP (10.37% vs 27.34%) (25). On the other hand, Chitsazi *et al*. concluded that both groups (test and control) showed a significant improvement in clinical parameters without observed superiority between the two methods (26). The mean PPD three months after therapy was 4.29 mm and 4.54 mm for the PDT and SRP group respectively. As far as CAL is concerned, in the test group the mean value was 5.29 mm, while in the control was 5.50 mm. The control group demonstrated significantly higher reduction in BOP compared with the PDT group (37.50% vs 75% mean BOP after three months) (26).

- PDT + SRP vs. SRP + antibiotics

Both parallel groups (PDT and antibiotic) had similar clinical parameters at baseline except for full mouth BOP (27,28). Control group exhibited higher mean BOP (74.2%) than PDT group (52.4%). PPD, CAL and BOP showed statistically significant improvement in both groups after three and six months of the provided therapy. Higher reduction of mean PPD and greater CAL improvement were observed in the antibiotic group after three and six months compared with the PDT group. The mean PPD of PDT group was 4.0 mm after three months and 3.9 mm after six, whereas the antibiotic group exhibited mean PPD of 3.2 mm and 3.0 mm after three and six months respectively. CAL was 4.7 mm for the PDT group in both follow-up measurements, while it was 3.9 mm and 3.6 mm for the antibiotic group after three and six months respectively. Mean BOP was similar between groups after both time periods (27,28).

- PDT vs. SRP alone

The authors of the single study concluded that both groups showed statistically significant PPD reduction and CAL gain in three months after therapy, but none of the treatment arms showed better clinical outcomes than the other (29). More specifically, the mean PPD for the PDT group was 3.49 mm, while the SRP group measured with 3.98 mm after three months. The adopted definition “relative clinical attachment level (RCAL)” was 8.74 mm (PDT) and 9.01 mm (SRP) three months after therapy. Non-significant were also the differences between the two groups regarding BOP after therapy (29).

## Discussion

- Summary of evidence 

The present study aimed to determine the effect of PDT on the non-surgical periodontal therapy of AgP. The results of this systematic search of the literature showed that only a few randomized controlled clinical studies had been performed to evaluate the effect of PDT in the therapy of AgP. Therefore, it was impossible to perform a meta-analysis of the existing data, because of the heterogeneity in methodologies of the included studies as well as the lack of publications. The search of the literature revealed that PDT has been utilized either as an adjunctive therapy (25-28) or mono therapy (29). The clinical outcomes of the PDT and SRP were compared with those of SRP alone (25,26) or SRP in conjunction with antibiotics (27,28).

In four of the included studies the use of PDT did not improve statistically significant the clinical parameters after non-surgical therapy of AgP (26-29). On the contrary, two of the included publications showed that three and six months after therapy with SRP and antibiotics, the clinical outcome was greater than SRP and PDT (27,28). It should be also noted that both treatment arms resulted in significant improvement in the total number of pockets ≥ 7 mm. Considering the pivotal role of residual deep pockets in the long-term prognosis of the periodontal diseases and the effect on further attachment and tooth loss (30), the greater reduction in the antibiotic group is more significant than in the PDT group. However, due to the lack of studies, the evidence is not yet strong enough (27,28). The results of these two publications confirmed the results of the meta-analysis of Keestra *et al*. which aimed to identify the effectiveness of systemic antibiotics as an adjunct to SRP in patients with untreated AgP (7). Systemic antibiotics in conjunction with non-surgical periodontal therapy exhibited a significant improvement in clinical periodontal parameters. Metronidazole and amoxicillin had a more clear benefit in the treatment of AgP than the other antibiotics (7).

Similarly, the results of another included study that utilized PDT in conjunction with SRP without antibiotic control group revealed that PDT had no additional benefit in the treatment of AgP (26). Contrariwise, the control group (SRP alone) demonstrated statistically significant greater elimination of BOP compared to test group (SRP+PDT). This outcome is rare in the literature either in the therapy of aggressive or chronic periodontitis (9,12,25-29). The repeated application of PDT during the periodontal therapy in several sessions may be crucial for improvement of the clinical parameters.

Only one study (25) revealed a beneficial role of PDT in conjunction with SRP in comparison with SRP alone, in which repeated application of PDT in four sessions was applied. The authors of this double-masked, split mouth randomized controlled clinical trial concluded that the PDT group showed greater clinical improvement in deep periodontal pockets three months after therapy than the control group. Low number of deep periodontal pockets eliminate the risk for progression of periodontal disease, and ensure the long-term periodontal stability (31).

In the included study that used PDT as a monotherapy, a statistically significant improvement of clinical parameters was identified after both treatments (SRP or PDT) (29). None of them exhibited higher beneficial role than the other. Although SRP represents an evidence based method to reduce bacterial load (32), specific periodontal pathogens such as *P. gingivalis, P. intermedia/nigrescens, and A. actinomycetemcomitans* may need additional therapy to be predictably eradicated (33). The effectiveness of mechanical treatment might be limited in inaccessible areas such as root concavities or furcations resulting in the need for a new protocol for non-surgical periodontal therapy. PDT on the other hand has the ability to destruct bacteria in a short period of time (under 60 seconds), and the risk of bacteria resistance is reduced (34). The results of the included study emerged PDT as a promising non-surgical therapy of AgP for the future (29). Additionally, PDT eliminate the discomfort, reduces the need for local anesthesia and might also be less time-consuming than scaling and root planing (35,36).

- Limitations and future studies

This systematic review showed that only limited randomized controlled clinical studies are available in the literature to assess the use of PDT in the treatment of AgP either as a mono therapy or in combination with SRP. The high heterogeneity between the included studies in regards to the study methodology did not allow us to conduct a meta-analysis. In addition, another limitation of the included studies was the short-term follow-up. The majority of the studies monitored the individuals for three months, while only one publication included six months evaluation period. In the future, more methodologically sound randomized clinical trials should evaluate the effectiveness of PDT in the therapy of AgP in order to obtain more reliable results. In addition, longer follow-up periods are required in the future. Furthermore, the potential role of several confounders such as genetic susceptibility, smoking, diabetes mellitus or alcohol consumption should be examined. PDT has been proposed as noninvasive alternative treatment option with clinical and microbiologic improvements during supportive periodontal therapy (treatment of residual pockets) when used as a mono therapy (36).

## Conclusion

Within the limitations of this review, PDT may exhibit a beneficial role in the therapy of aggressive periodontitis after repeated applications. The PDT as a mono therapy has similar significant clinical outcomes with the mechanical therapy. However, the antibiotic coverage with amoxicillin and metronidazole in conjunction with SRP seems to have a greater impact in the clinical outcomes after therapy compared to SRP and PDT. In the future, more methodologically sound, long-term randomized clinical trials are needed to be conducted aiming to examine the effectiveness of this non-surgical periodontal therapy.
